# Event-Based Computation of Motion Flow on a Neuromorphic Analog Neural Platform

**DOI:** 10.3389/fnins.2016.00035

**Published:** 2016-02-16

**Authors:** Massimiliano Giulioni, Xavier Lagorce, Francesco Galluppi, Ryad B. Benosman

**Affiliations:** ^1^Department Technology and Health, Istituto Superiore di SanitàRome, Italy; ^2^Vision and Natural Computation Group, Institut National de la Santé et de la Recherche MédicaleParis, France; ^3^Sorbonne Universités, Institut de la Vision, Université de Paris 06 Pierre et Marie Curie, Centre National de la Recherche ScientifiqueParis, France

**Keywords:** spike-based, motion-flow, analog VLSI, silicon retina

## Abstract

Estimating the speed and direction of moving objects is a crucial component of agents behaving in a dynamic world. Biological organisms perform this task by means of the neural connections originating from their retinal ganglion cells. In artificial systems the optic flow is usually extracted by comparing activity of two or more frames captured with a vision sensor. Designing artificial motion flow detectors which are as fast, robust, and efficient as the ones found in biological systems is however a challenging task. Inspired by the architecture proposed by Barlow and Levick in 1965 to explain the spiking activity of the direction-selective ganglion cells in the rabbit's retina, we introduce an architecture for robust optical flow extraction with an analog neuromorphic multi-chip system. The task is performed by a feed-forward network of analog integrate-and-fire neurons whose inputs are provided by contrast-sensitive photoreceptors. Computation is supported by the precise time of spike emission, and the extraction of the optical flow is based on time lag in the activation of nearby retinal neurons. Mimicking ganglion cells our neuromorphic detectors encode the amplitude and the direction of the apparent visual motion in their output spiking pattern. Hereby we describe the architectural aspects, discuss its latency, scalability, and robustness properties and demonstrate that a network of mismatched delicate analog elements can reliably extract the optical flow from a simple visual scene. This work shows how precise time of spike emission used as a computational basis, biological inspiration, and neuromorphic systems can be used together for solving specific tasks.

## 1. Introduction

Take a pen, move it in front of an optical sensor and try to state, from the sensor output, its direction, and speed. How can we extract information about its motion? And how can we encode and communicate this information? It is an interesting problem related to simple questions. Answering these questions would pave the way toward the design of smart sensors performing object segmentation (Tian and Shah, 1996; Zitnick et al., 2005), autonomous robot navigation and obstacle avoidance (Nelson and Aloimonos, 1989; Srinivasan and Gregory, 1992; Ilic et al., 1994; Camus et al., 1996; Coombs et al., 1998). It is therefore not surprising that a large amount of scientific effort has been devoted in trying to answer them, using different approaches.

Traditional ways to solve this problem rely on off-the-shelf hardware and start with freezing the visual scene in a sequence of fixed frames. Frames are then analyzed to obtain the visual flow information. Various algorithms exist to perform this task: some estimate the flow using velocity-tuned filters designed in the Fourier domain (Watson and Ahumada, 1983; Heeger, 1987, 1988); others exploit phase-based methods Barron et al. (1994); many compute correlation indexes or search for matching regions in adjacent frames (Sutton et al., 1983; Kories and Zimmermann, 1986; Anandan, 1989; Singh, 1991; Camus, 1995; Banks and Corke, 2001); other methods use spatio-temporal derivatives of image intensity under the constraint of a constant brightness (Horn and Schunck, 1981; Nagel, 1987; Galvin et al., 1998). All these methods are tied to frames, and to the frequency at which they are captured. Their high computational cost makes embedding them on compact devices for real-time applications challenging. Nevertheless, noticeable examples have been published (Mehta and Etienne-Cummings, 2003; Grue and Etienne-Cummings, 2004). They partially rely on the computational power of a dedicated microcontroller to extract the optical flow using simplified versions of gradient-based methods.

Biological systems rely on different operating principles: information is not represented in frames, but by means of data-driven pulsed messages exchanged by complex nervous cells; information processing is not performed algorithmically, but supported by specific neural circuitry. For example, Barlow and Levick (1965) demonstrated that an inhibitory mechanism is at the basis of the computation the biological retina performs to extract the direction of motion of an object in the visual field. Inspired by these studies, we hereby present an architecture that does not rely on capturing and processing frames. We make use of neuromorphic retinas (Mead and Mahowald, 1988; Culurciello et al., 2003; Culurciello and Andreou, 2006; Lichtsteiner et al., 2008; Delbruck et al., 2010; Posch et al., 2011; Serrano-Gotarredona and Linares-Barranco, 2013): they are frame-free devices whose pixels, each independently and asynchronously, can directly communicate with the next processing stage without having to wait for a global synchronization step that collects all their output in a frame. By doing so the precise timing at which a pixel is activated becomes a computational variable which is readily available. Neuromorphic pixels react only to light changes and are blind to a steady state of illumination, in analogy with ganglion cells, their biological counterparts. Since motion induces sparse spatio-temporal activity, retina pre-processing opens the way to lighter methods of analysis (Bauer et al., 2007; Serrano-Gotarredona et al., 2009; Clady et al., 2014) more suited for hardware implementations in smart sensors.

Here we present a neural architecture that receives the retina output and computes the optical flow with milliseconds latency. This work is a neural implementation of the event-based optical developed in Benosman et al. (2014), not surprisingly the implementation matches the original work of Barlow and Levick (1965) on the direction-selective ganglion cells in the rabbit's retina. Our neuromorphic architecture provides possible answers to both the questions posed above on how the motion information can be extracted and how it can be communicated in hardware. The specific goal of this work is to use a neural architecture inspired by the motion estimators found by Barlow and Levick, implement it using analog VLSI neurons to extract direction and speed estimation on specific scenarios in neuromorphic hardware.

The next Section reviews previous approaches to extract motion information, using standard computers or neuromorphic hardware. Section 2.2 describes the system architecture and the components used, presenting a robust processing method, tolerant to noise and parameters' mismatch. In Section 3 we demonstrate the performance of our approach by running increasingly complex experiments. We finally discuss its limitations and propose some future developments in Section 4.

## 2. Materials and methods

### 2.1. Review of neuromorphic motion sensors

In 1986 Tanner and Mead (Tanner, 1986; Tanner and Mead, 1986) presented their first compact neuromorphic motion detector. They designed a photosensitive matrix including analog circuits implementing a gradient-based algorithm in every pixel (Horn and Schunck, 1981). These systems suffered from low accuracy in divisions performed with analog circuitry. Nevertheless, their results boosted the field and various groups adopted the same approach in the subsequent years (Deutschmann and Koch, 1998; Stocker and Douglas, 1998; Stocker, 2004). In particular in Stocker (2004) the author describes a two layer recurrent network which performs object segmentation and solves the aperture problem. They rely on the Horn and Schunck equations and on a smoothness constraint to compute the optical flow. They do so by separating an input layer which uses an array of photoreceptors to compute the derivatives of the image brightness, and feeds them to a second layer, a resistive network, which imposes the smoothness constraint. The output of their system is a set of analog values read by a scanning circuitry.

The drawback of gradient-based algorithms is that they assume a constant illumination. An alternative solution is offered by the motion detection system studied in 1956 by Hassenstein and Reichardt (Hassenstein and Reichardt, 1956). They proposed an Elementary Motion Unit (EMU) which correlates illumination levels recorded at different instants and positions of the image. To implement EMUs only photo-pixel, delays and multipliers are required, and multipliers can even be substituted with other forms of comparator simpler to design (Gottardi and Yang, 1993; Harrison, 2005; Pant and Higgins, 2007).

Using the adaptive retina photopixels as the front-end for the EMUs, correlation takes place between light-changes rather than on absolute light intensities. This solution guarantees sensitivity over a wide range of illumination levels and simplicity in the implementation. For these reasons this solution is one of the most widely adopted (Horiuchi et al., 1990; Andreou et al., 1991; Delbruck, 1993; Etienne-Cummings et al., 1993; Meitzler et al., 1993, 1995; Harrison and Koch, 1999; Liu, 2000).

Based on delays and correlation, the units proposed by Reichardt are translated into a particular speed value to which they respond maximally. By changing the correlation method and its parameters the speed selectivity range of an EMU can be tuned from just a narrow to a wide range of speeds (Kramer and Koch, 1997). In other words, by increasing the range it is possible to create an EMU measuring the time a light variation takes to travel from a pixel to another. Various circuits have been studied to explicitly compute the time-of-travel of the light-change. They are all based on similar mechanisms: when a light-change is firstly detected, a facilitation signal starts a sort of analog counter, typically a decaying voltage trace; when the light-change is detected by the second pixel, the decaying trace is directly read (*Facilitate and Sample*; Kramer and Koch, 1997; Indiveri et al., 1999; Möckel, 2012) or compared with another signal (*Facilitate and Compare*; Deutschmann and Koch, 1998) to provide the time-of-travel. In Kramer and Koch (1997) and Kramer (1996) the authors also propose to encode the time-of-travel in the duration of a pulse determined by the activity of adjacent photopixels.

Rather than using Reichardt detectors, in Arreguit et al. (1996), the authors use a random pattern (on a mouse ball) to subsequently activate an array of photodiodes; the difference of activation of neighboring photodiodes gives the direction of movement, while the magnitude of the speed is given by the number of photodiodes responding to moving edges. This system relies on a particular—rather than general —scene to be analyzed; it will hardly estimate the speed of a single point, with the same size of a photodiode, displaced across the photodiode array.

In these above the output of motion detectors is directly read via dedicated access points or through *ad-hoc* scanning systems. The issue of accessing all the extracted motion information at every pixel location is not discussed in depth. The reason is that the authors either focus on implementing a single motion-detector or present their devices as ego-motion detectors, where a single global averaged value should be provided as output.

**Figure 1:**
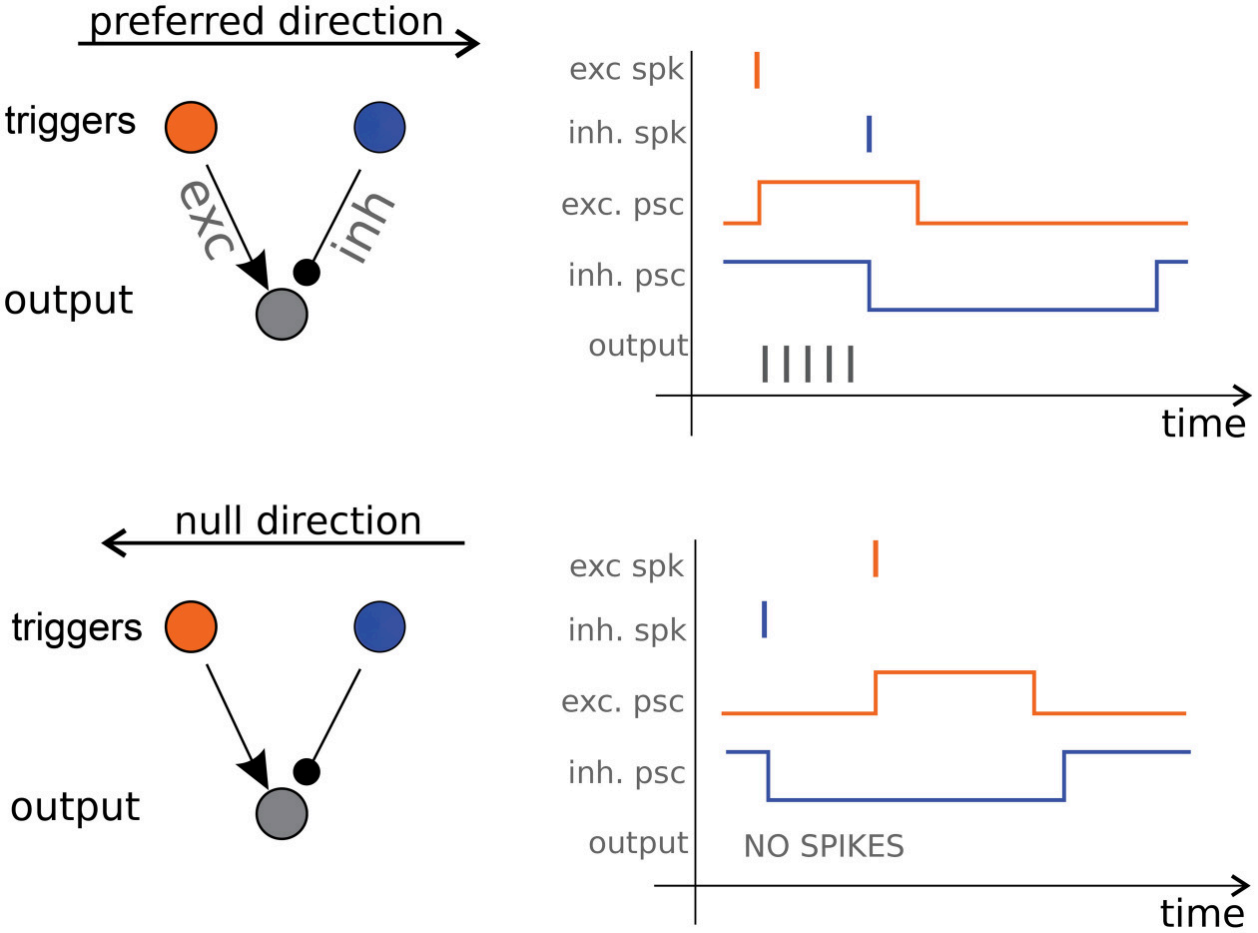
**Direction Selective cell**. On the left panels the three-neurons architecture consisting of a *start trigger* (the excitatory orange neuron) a *stop trigger* (the inhibitory blue neuron) and the output *counter* neuron (in gray). Excitatory and inhibitory post-synaptic current pulses cause the output neuron to fire only when the object is moving in the preferred direction (right panels).

It is possible to draw an analogy between some artificial and natural motion detection systems. Barlow and Levick (1965) described a pulse-based mechanism similar to the one proposed by Kramer (1996) where direction selectivity derives from lateral asymmetric inhibition pulses. Benson and Delbrück (1991) reports a fully analog device based on this idea. Barlow and Levick proposed their inhibition-based scheme to explain the activity of the Direction Selective (DS) ganglion cells in the rabbit's retina. In their model the pulses coding for speed are post-synaptic currents that induce firing activity in the DS ganglion cells. Their scheme requires just three neurons (see Figure 1, left panel): two *triggers*, a *start* and a *stop* one, and an output *counter* (which corresponds to the DS cell). A spike emitted by the *start* neuron excites the *counter* which starts firing until a spike from the *stop* neuron inhibits it (see Figure 1, right panels). The number of spikes emitted by the counter is proportional to the start-stop delay. If the duration of the excitatory pulse is much shorter than the length of the inhibitory one, this simple unit becomes selective, up to a certain delay, to the sequence of *trigger* activation, e.g., to the direction of motion.

With this scheme Barlow and Levick answer our question on how motion information can be coded and extracted, and also provide an answer to our second question, on how such information can be communicated: they are proposing asynchronous spiking communication over the optical nerve. Their DS units are the core of our system: we use a silicon retina to provide an input to the DS units and we obtain motion information from the output spike trains. The complete architecture is described in details in the next Section.

### 2.2. Information processing architecture

Our proposed architecture comprises three neuronal layers arranged in a feed-forward fashion: retinal neurons, edge-detectors neurons, and output *counter* neurons. To prepare our information processing chain we followed these steps: we grouped the retinal pixels in a grid of macropixels, each sensing a different area of the visual field; we connected each macropixel to a different neuron to obtain robust edge-transit detectors. We added a layer of output neurons (the direction sensitive ganglion cells, or DS) and we followed the Barlow and Levick model for their afferent connections to obtain direction selective units. We tested the entire chain under controlled conditions and tuned neuronal and synaptic parameters as well as macropixels dimension and position to obtain reliable motion extraction over the desired range of speeds.

**Figure 2:**
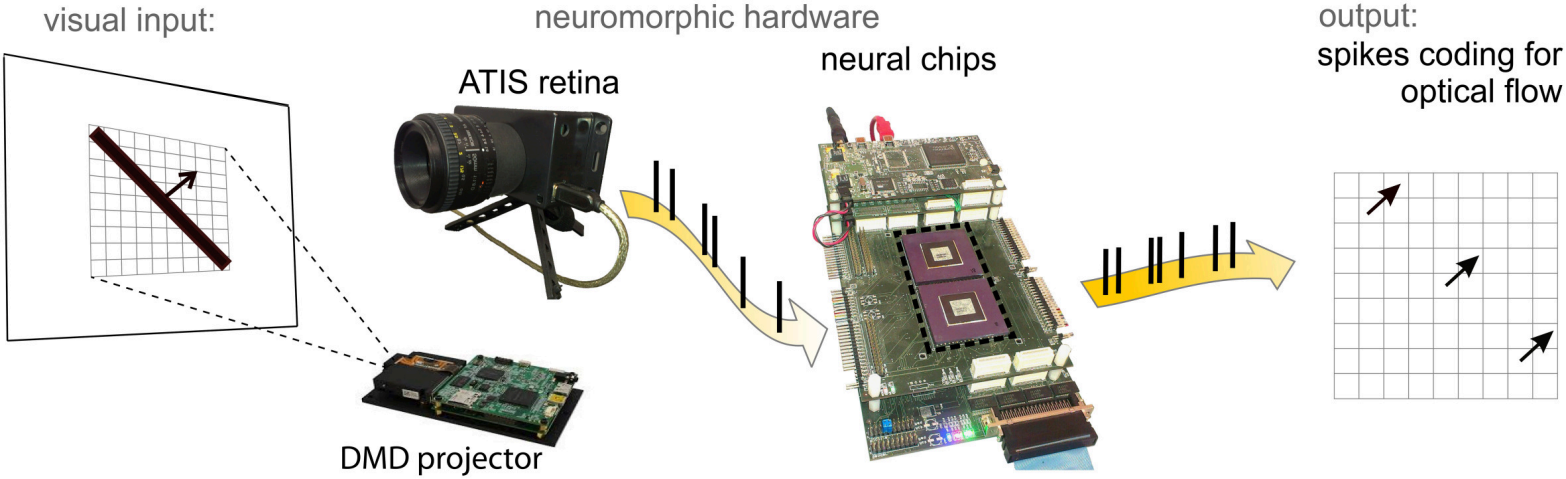
**ATIS neuromorphic retina in front of a screen with a moving bar projected by the DMD**. Spikes from the retina are fed into the neural chips (central panel) hosting a 3 × 3 matrix of motion detectors. The network output is monitored by a PC for on- and off-line data visualization.

Figure 2 shows the hardware setup hosting the architecture. The following paragraphs discuss in details the crucial aspects of this information processing chain.

### 2.3. Retinal layer

As a visual front-end we use the ATIS retina described in Posch et al. (2011). It is a matrix of 304 × 240 photopixels of which we use just a central part of 45 × 45 pixels. In addition to adaptive, contrast-sensitive photopixels, the ATIS retina can also measure absolute levels of illumination. However, we do not use this feature in this work. Every adaptive photopixel is connected with two neurons, one emitting events (spikes) only when illumination increases, one firing when illumination decreases. Up to a certain limit, larger or faster light changes cause the emission of bursts with higher spike frequencies. In this work we exploit only those neurons encoding for dark-to-bright transitions, which react to the passage of the leading edge of a bright moving bar. Our retina is placed in front of a screen, pointing at bright moving bars. Its output is transmitted to two neuromorphic chips (see Figure 2), dubbed FLANN (Final Learning Attractor Neural Network (Giulioni et al., 2008) and described in the following paragraph. The bar is projected on the screen using a Digital Micromirror Device (DMD) by Texas Instruments which ensures a frame rate of 1 kHz, so as to avoid temporal artifacts on the retina output.

The output from the silicon retina is not perfectly homogeneous over the entire matrix. Its analog circuits are subject to parameter mismatch that derives from imperfections in the manufacturing process. Therefore, some pixels may be highly sensitive, while others may be hard to activate. Noise on photodiodes is another source of variability which, along with mismatch, frequently causes many false-positive detections. And false negatives are also possible.

To reduce edge detection errors, we group single retina pixels in macropixels of 5 × 5 pixels each, and we convey the spiking activity of each macropixel onto a single FLANN neuron. As explained in the next section, this neuron acts as a robust edge detector.

### 2.4. FLANN chip

FLANN chips are described in details (Giulioni et al., 2008). Each chip accommodates 128 neurons with 128 synapses per neuron. FLANN neurons are integrate-and-fire ones endowed with linear decay (β) and a lower bound (*V*_*min*_) for the membrane potential *V*. They implement in hardware the model described in Fusi and Mattia (1998):

$$\begin{array}{ll} & C\dot{V}\left(t\right)=-\beta +I\left(t\right)\;\beta \geq 0 \end{array}$$

$$\begin{array}{ll} If & V\left(t\right)\leq V_{min},\text{then }V\to V_{min} \end{array}$$

where *C* is the membrane capacitance, *I*(*t*) is the total afferent synaptic current. When *V*(*t*) = θ a spike is emitted and *V* resets to *V*_*min*_. Upon the arrival of a pre-synaptic spike synapses generate rectangular post-synaptic currents. Synapses also have an internal dynamics designed for Hebbian plasticity that, for this work, has been disabled. The synaptic matrix can be reconfigured at will to implement different kinds of networks.

FLANN chips host the DS units: one chip host the *triggers* the other one hosts the output *counters*. Every *trigger* receives spikes from a different retina macropixel and acts as a robust visual edge detector. To obtain robustness the neuronal and synaptic parameters have been tuned such that a trigger reaches its firing threshold only when at least five retinal pixels fire within a small time period. β is tuned to 3.5±0.5 in units of [(θ−*V*_*min*_)∕*s*] and the efficacy of synapses is 0.20±0.05 in units of [θ−*V*_*min*_]. Regarding the *trigger* neurons we found convenient to exploit their analog properties to filter out noise.

Put differently, in the macropixel-to-trigger connection, we are using redundancy and averaging, together with the neuronal non-linearity, to reduce both wrong and missed detections. A convergent many-to-one connection seems to be adopted also by biological systems in the inner retina layers (Masland, 2001), although in this case cells interact through analog signals, rather than using spikes. In our implementation spikes are more convenient, since retinal neurons and DS units are hosted on different chips, and inter chip communication is more efficient with spikes (in the form of digital asynchronous pulses) than with analog values.

### 2.5. Asynchronous spike communication

Spike communication amongst chips preserves the precise spike timing which is at the basis of our computational architecture. Off-chip communication is handled through an asynchronous protocol named Address-Event Representation (AER; Boahen, 1999) which, in regimes of sparse activity, guarantees high timing accuracy by encoding time in the occurrence of the event itself. The output of the retina is thus an asynchronous and continuous flow of data. It encodes implicitly the time of a light variation, and explicitly, in the neuronal addresses, the light-change location.

Communication along this feed-forward chain is mediated by the PCI-AER board (Dante et al., 2005; Chicca et al., 2007) which, in hardware, maintains the mapping table for the connectivity. The maximum transmission latency on the AER bus is half a microsecond per spike, absolutely negligible in our experiments. The same board lets us monitor the AER traffic, also in real-time. We use this feature for on-line visualization of the optical flow.

### 2.6. 2D motion detectors

**Figure 3:**
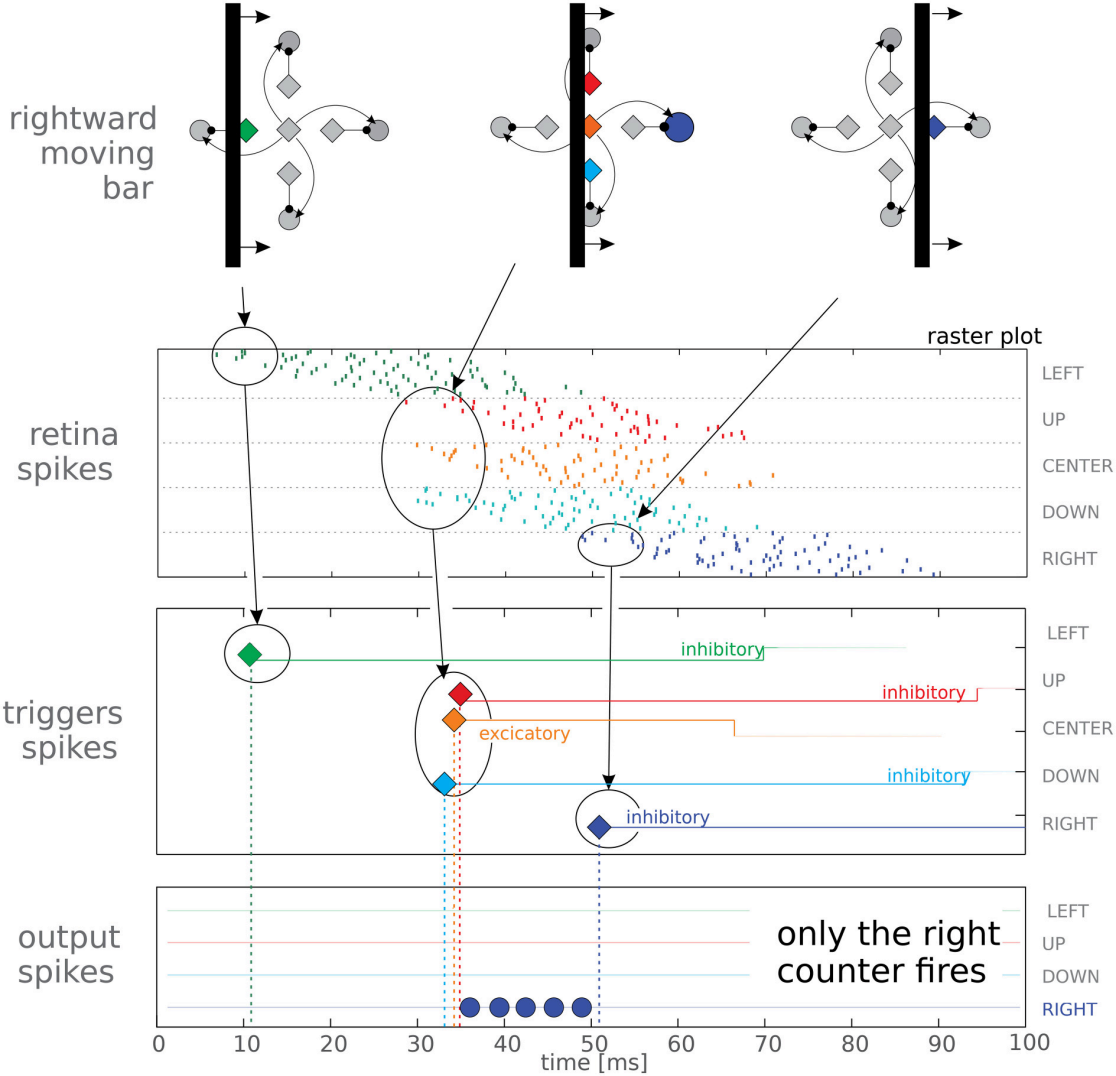
**Top panel reports a schematic view of a vertical bar moving rightwards, and reports the logical organization of the macropixel connectivity**. The other panels show the spiking activity elicited in the three layers (retina, triggers, and output counters) of our feed-forward network. The last two panels represent spikes from the trigger and output neurons, represented as diamonds and circles, respectively; different orientations have been coded with different colors. It can be noted that the red trigger fires after only 3 spikes, due to mismatch. In the trigger panel we also report the duration of the post-synaptic currents after each spike, lasting, in this test, 60 ms for the inhibitory neurons and 34 ms for the excitatory one. In the lower panel we report data from only 1 neuron per output counter (in fact we use 3 neurons per counter to average out process mismatch, see text for details); correctly only the rightwards output neuron gets activated.

A single direction sensitive (DS) unit provides information only if the object moves in its preferred direction (see Figure 1), otherwise it stays silent. To extract a 2D time-of-travel vector we combined four DS units together in a single motion detector (see Figure 3, upper panels). The four DS units share the same *start* (orange) neuron while they have four different *stop* neurons mapped onto the retina macropixels such that they are selective to upwards, downwards, leftwards, or rightwards movements. It is worth noting a four-directions system is used in biological systems as a valid complete base for focal plane motion extraction (Masland, 2001). Correspondingly, each 2D motion detector has four output *counters*. In our implementation, rather than using just a single neuron as *counter*, we use a set of three neurons per *counter*. This redundancy helps to reduce the effects of mismatch due to fabrication imperfections in the analog circuitry. Synaptic and neuronal parameters are set such that excitatory currents make counters fire regularly at about 300 Hz while inhibitory currents act as shunting inhibition. Our complete system comprises nine motion detectors organized in a 3 × 3 grid.

## 3. Results

### 3.1. Single motion detector

In order to characterize the basic units of our system, we projected a vertical bar moving rightwards on the DMD screen. A schematic view of the moving bar is presented in the top panel of Figure 3; the Figure shows different time instants to characterize how different neurons are activated as the bar is passing. The top panel shows a logic representation of the connectivity of the macropixel rather than a geometric one; in reality the excitatory and inhibitory neurons are arranged in two parallel populations and deployed in different chips. The elicited network activity for a single motion detector is reported in the raster plots of Figure 3. Spikes from retinal neurons are grouped according to the emitting macropixel. The last two panels represent spikes from the trigger and output neurons, represented as diamonds and circles, respectively; different orientations have been coded with different colors. As time goes on, spiking activity shifts toward different neurons, following the leading edge of the moving bar at time *t* = 35 ms the excitatory trigger emits a spike and consequently the rightward counter, and only that counter, starts firing until the inhibitory stop trigger, shown in blue, blocks it. This demonstrates, in hardware, the correct behavior of the basic building block of our network. As stated above, we make use of three neurons per counter (in Figure 3 only one neuron per counter is shown for easy visualization). Frequently mismatch derives from fabrication imperfections. To reduce this discrepancy, if necessary, the counters' outputs could be averaged in a successive computational stage.

Triggers emit just a single spike, and then enter a long absolute refractory period in which they are insensitive to afferent currents. Each trigger extracts a single bit of information from an incoming spike. It also filters out the noisy, false positive spikes as explained above, and prevents the counter activity from global variations in the retina output. The activity of the pixels is affected, although only slightly, by the room temperature, by the level of steady illumination and by the speed of the bar. Regarding triggers, the working conditions of the counters remain always the same and this greatly simplifies the tuning of their parameters. Counters could still work even if the triggers were to be removed, but their tuning would be more challenging.

The use of a vertical or horizontal bar with DS units oriented in the XY directions creates a critical condition for the system: at *t* = 35 ms (in Figure 3) the shared start trigger fires almost simultaneously with the the upward and downward stop neurons. They do not fire exactly at the same time because of the noisy retina activity and of the mismatch. If one of the two *stop* triggers arrives too late, the corresponding counter fires at least one spike, signaling a very short delay (i.e., very high speed) in the wrong direction. To avoid this effect, parameters need to be set such that a certain amount of misalignment can be tolerated. This boils down to two constraints: (1) the firing rate of the counter should not be too high, thus guaranteeing a *safe* latency time before the first spike emission (about 3 ms in our case), and (2) inhibitory post-synaptic currents should ensure a rapid shunting effect, which means that the rising edge of the post-synaptic current should be fast, as it is for our rectangular current pulses. Apart from these technical details, this critical condition also suggests to remain in the domain of time-of-travels when performing further computation. When moving to the domain of speeds, a single wrong spike could mean the highest possible evaluation error.

### 3.2. Optical flow in the 3 × 3 detectors grid

**Figure 4:**
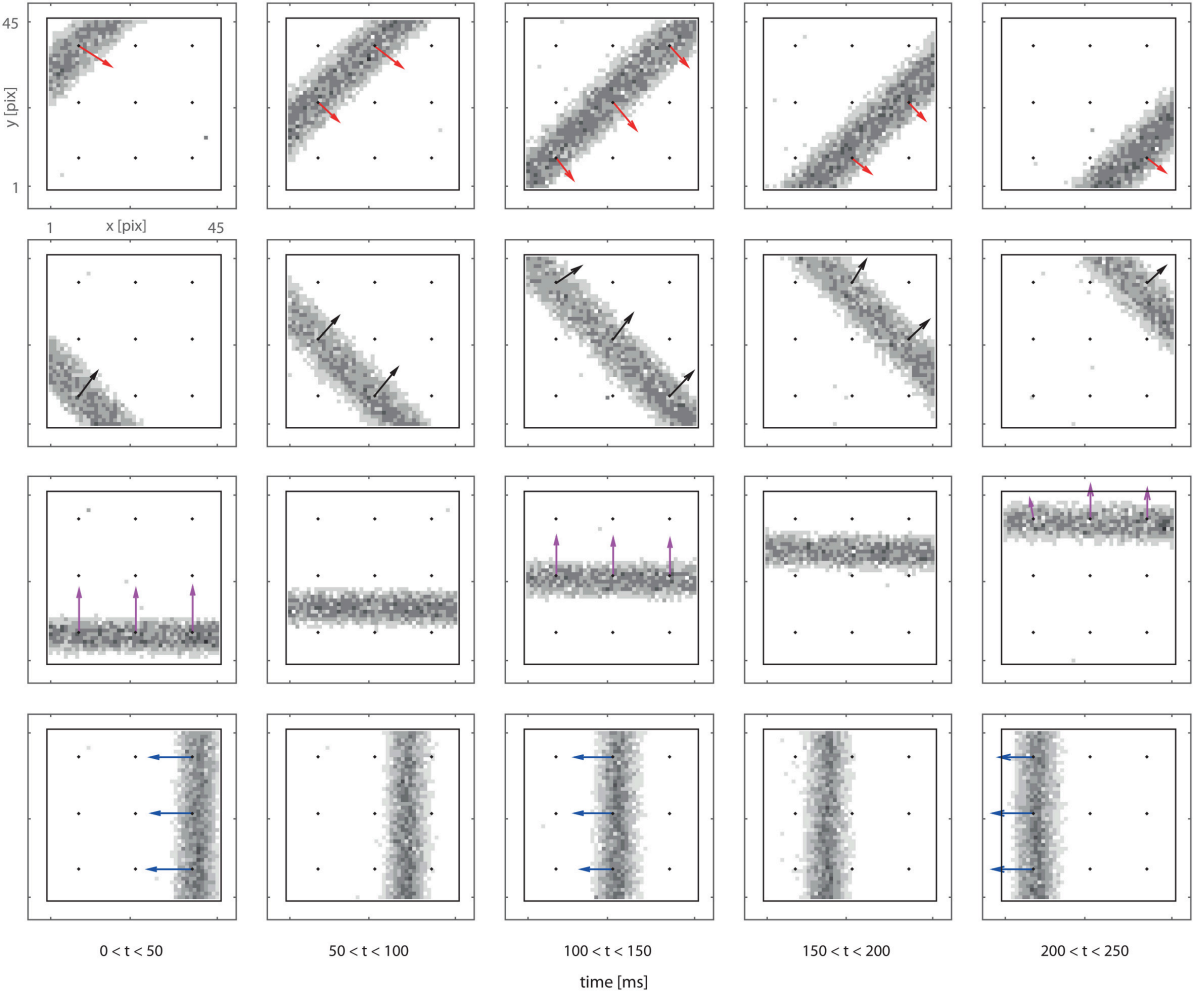
**Delay vector fields (colored arrows) overlapped to retina spiking activity accumulated over 0.2 s (in grayscale)**. Black dots in the figures mark the locations of the DS detectors. Arrows are reconstructed from the spiking activity of the output counter neurons (see text). The visual input consists in a single passage of a bar moving perpendicularly to its axis. Every row of panels refers to a different bar orientation. Each frame is built using all the spikes received in a 50 ms time window. The length of the arrow is proportional to the number of spikes produced by the detector.

We now consider a complete system comprising a 3 × 3 grid of motion detectors, and we expose the system to bars moving in different directions. Figure 4 presents both the retina output and delay vectors obtained from the counters' activity. Each row in Figure 4 corresponds to a different trial. In each trial the bar moves at a fixed speed in a given direction. The vector field is obtained from the off-line analysis of the recorded spike train. In the figure we plot our data in the form of a sequence of frames. Each frame is built using all the spikes received in a 50 ms time window. Even though a frame-based representation does not convey detailed information on the spike timing, it provides an intuitive visualization of our data which, we stress, are provided asynchronously, continuously and in real-time by our feed-forward network.

By monitoring the continuous stream of output spikes, the vector field can be computed and visualized in real-time. The only critical issue is defining when the measurement is completed. For our real-time tests we relied a successive approximation procedure. Consider the output of a single detector: upon the emission of the first spike, a first approximation of the time-of-travel vector can be plotted; the vector orientation and its length can then be further updated on the emission of other spikes. This successive approximation process should end when spikes are not fired for a period exceeding a certain threshold; in our real-time tests this period was safely set to 10 ms.

We stress that this method of data analysis relies only on the activity of our output neurons. All the necessary information is encoded in the bursts emitted by the counters, and spikes are only fired when a moving object is detected: the computation is data driven and redundancy is eliminated at the sensor level. A first estimation of the optical flow is available a few milliseconds after the passage of the object. By waiting a bit more, a more precise measure is obtained. This is another interesting feature of our system, which opens to different levels of approximation in successive elaboration stages.

**Figure 5:**
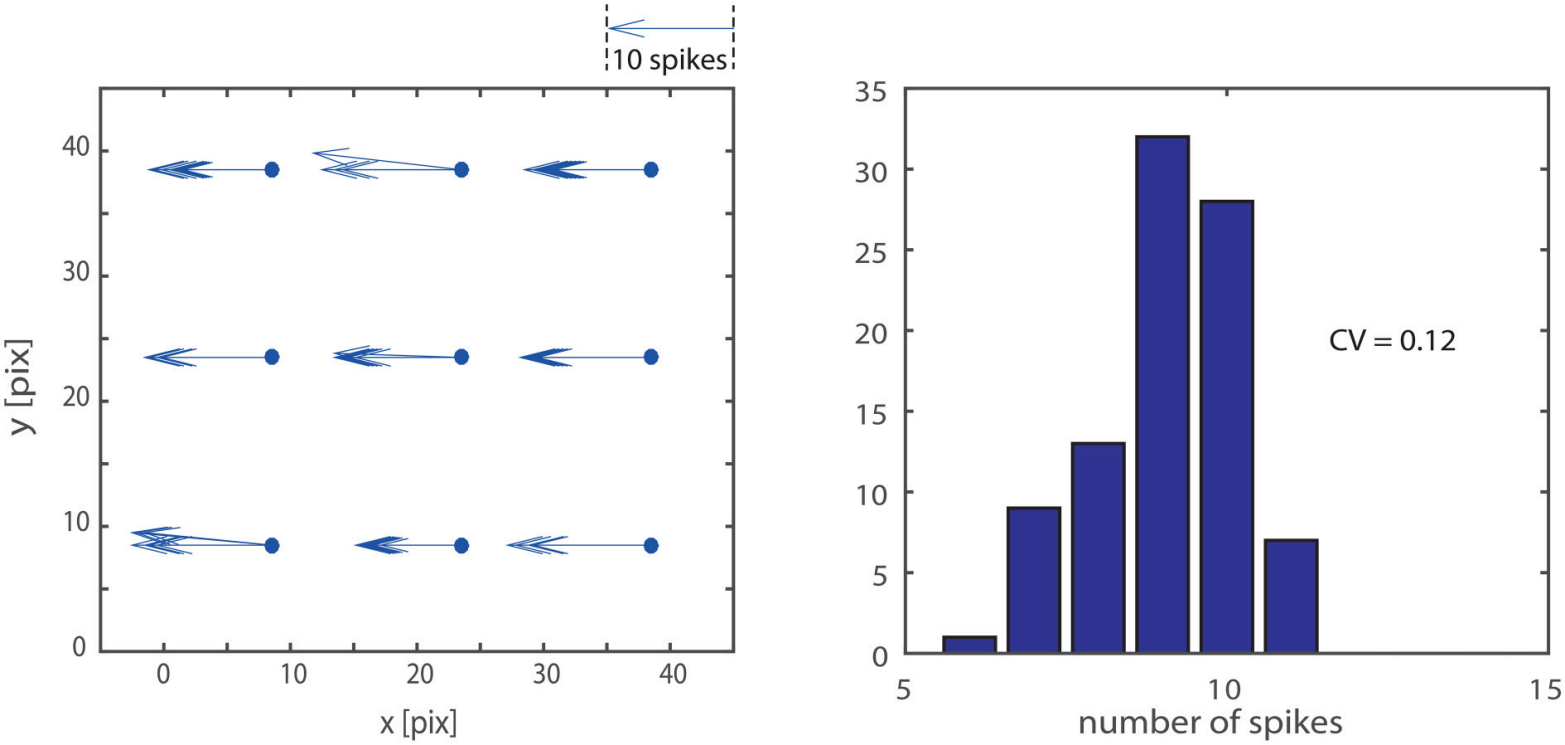
**Measurement repeatability: we stimulated the system 10 times with a vertical bar moving leftwards at a given speed**. The left hand panel shows an intuitive representation of the data, where each repetition across the nine detector is explicitly represented by an arrow; the length of the arrow is proportional to the number of spikes produced by the detector. The distribution of the nine detector outputs shows the accumulated data from different detectors and different passages of the bar. The overall CV is 0.12.

As visible in Figure 4 not all the delay-vector, from a single trial, have the exact same length and orientation. This is due to noise and mismatch. To evaluate the repeatability of the measurement we stimulate 10 times the system with a vertical bar moving leftwards at given speed. We accumulated the results in the left panel of Figure 5 which provides an intuitive visualization of the data. Quantitatively, for what concerns the absolute value of the measured delay, the CV of a single detector output, ranges from 0.05 to 0.12. The variability in a single detector output is mainly due to the noise in the retina output which is not completely removed by the macropixels-to-trigger convergent connections. Another important measure is the amount of mismatch among different detectors: considering a single passage of the bar, the distribution of the nine detector outputs, on average, have a CV of 0.12. In the right panel of the figure we accumulated data from different detectors and different passages of the bar. The overall CV is 0.12.

**Figure 6:**
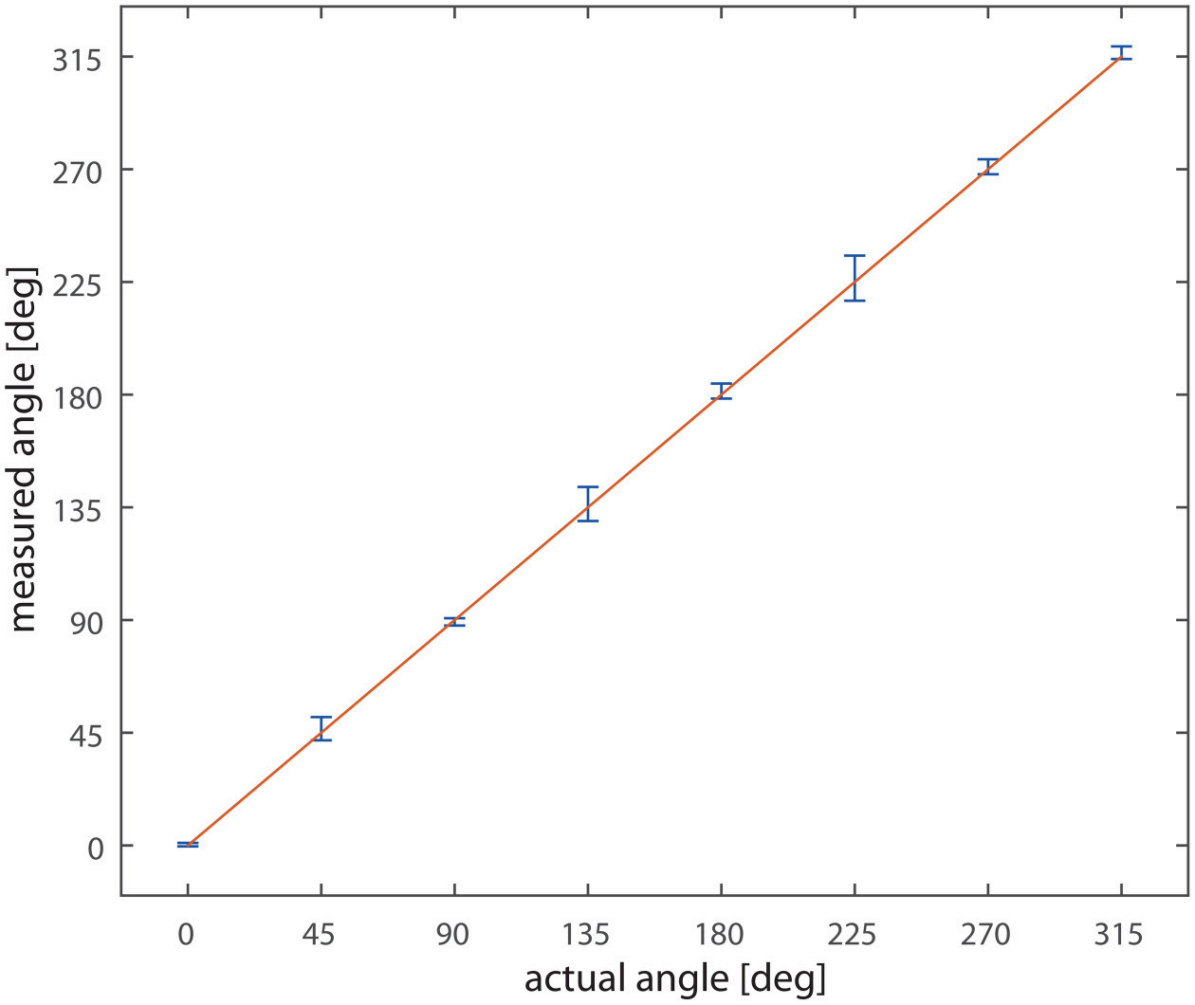
**Measured vs. actual angle**. On the x-axis the actual direction of motion of the bar, on the y-axis the measured value. Data are collected from the nine detectors. The line x = y is overlaid to the data. Bars represent the standard deviation.

Varying the direction of bar motion over 360° and recording network output to single passages of the bar we obtained the data plotted in Figure 6; the graph compares the actual direction of the bar with the detected one. The straight line and the small error bars demonstrate the level of reliability of the system; the highest error is below ±3°.

### 3.3. Motion amplitude detection

**Figure 7:**
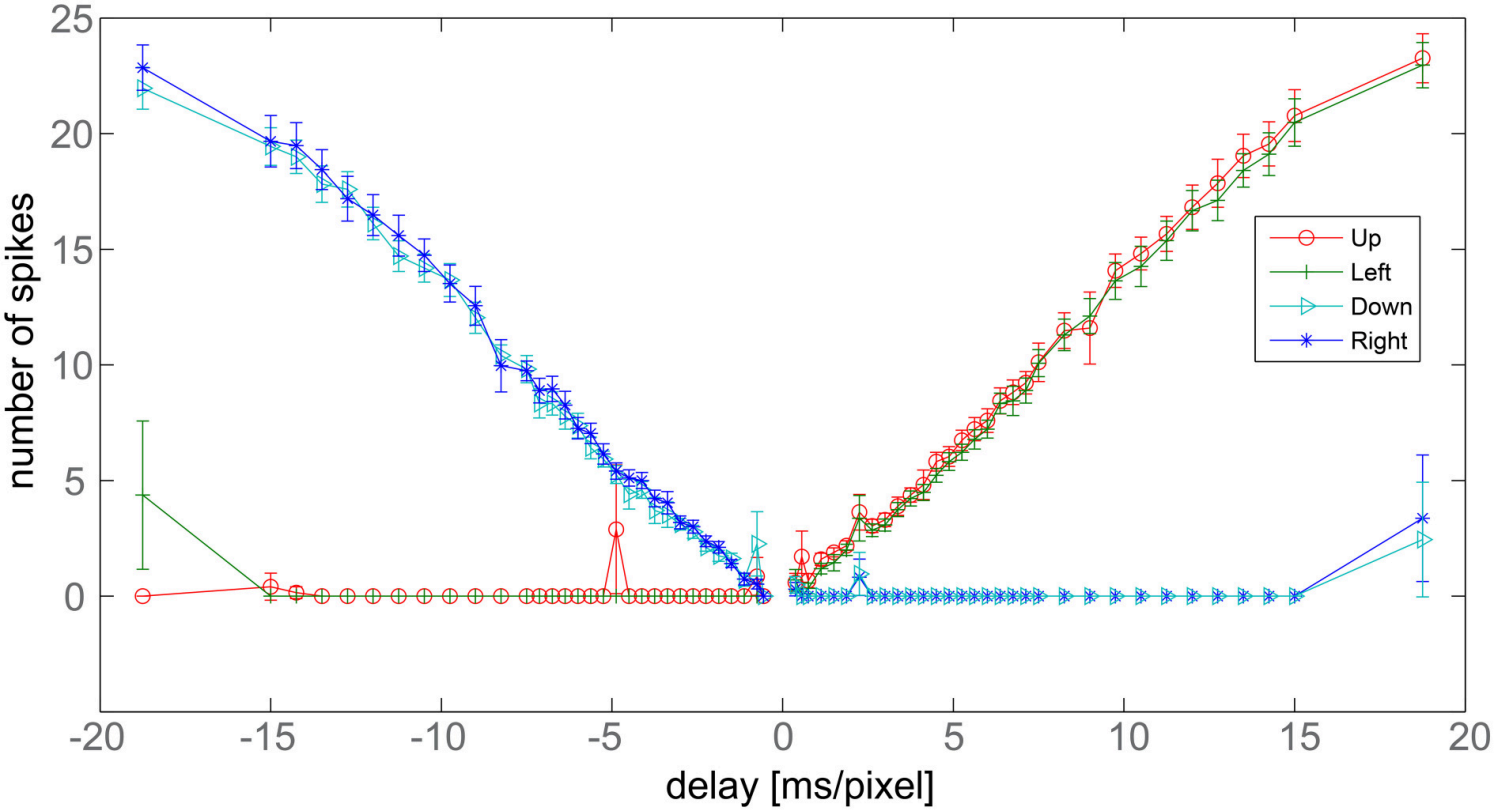
**Delay vs. number of output spikes**. Data refers to a bar tilted by 45° with respect to x-axis, moving perpendicularly to its axis. The x-axis reports the inverse of the bar speed. Negative values refer to bars moving toward the down-right corner, positive values correspond to bars moving toward the upper-left corner. The y-axis shows the average number of spikes fired by the nine detectors after a single passage of the bar at a given speed. Bars report the standard error.

We now focus on the response of the system to a bar moving at different speeds and tilted by 45°. When the bar moves toward the upper-left corner of the visual field, to obtain vectors tilted exactly by an angle of 45° with respect to the x-axis, upwards and leftwards counters should fire the same number of spikes. The average number of spikes emitted by the nine detectors, together with the corresponding standard deviation, is reported in Figure 7 for different bar speeds (the maximum coefficient of variation in the x-range 1.5–15 [ms/pixel] is 0.13). As expected, for positive speeds only the upward (red) and leftward (green) DS units detect the motion, while the other units do not emit spikes. Moreover, the red and green curves largely overlap, signaling a 45° apparent motion. The system works correctly although errors, due to noise, exist in some trials. We note here that a simple way to reduce the residual error, if necessary, would be to add an extra layer of averaging so as to have the nine detectors contributing to a single output. This extra averaging would be in line with biological data, where many sub-DS units converge on single DS ganglion cells (Masland, 2001).

The graph also shows a linear response of the detector. Linearity derives from the rectangular shape of the post-synaptic current pulses, from the reliability of the light-to-spike transduction operated by the retina and from the noise-filtering ability of the trigger neurons.

In Figure 7, the leftmost points show the major limitation of the system. We have spikes from right and downwards counters but also from the leftwards one. Undesired spikes are emitted because inhibition ends before the excitatory synaptic currents. The maximum delay correctly measured by the system equals the difference in lengths between the excitatory and inhibitory pulses. Spikes from opponent counters signal the limits of the system. We note that Barlow and Levick observed the same issue in the rabbit's DS ganglion cells (see the Section 4 for further details). Another limitation of the system can be observed for instance at −5 ms/pixel for the upward direction detector: this is the most serious limitation of our system happening when mismatch and noise combine together disrupting the trigger spike-timing.

### 3.4. Emulation of larger detectors grids

Our network architecture is fully asynchronous and parallel and the nine detectors act independently from each other. In other words the system is scalable. The maximum size of the detector grid is set by the number of neurons in our neural chips. In larger chips, a larger grid can be deployed without affecting the system performance. In the last two experiments we emulate a larger detector grid to show the scalability of the proposed approach. To obtain those vector fields we follow this procedure: (1) we record the output of the retina observing the entire scene, (2) we split the retinal spike train into 6 × 6 parts related to different scene areas, (3) we send, one-by-one, the retinal spike trains to the neural chips which compute, in real-time, the optical flow, and (4) we collect together all the output spikes to visualize the vector fields of the entire original scene in a single figure.

**Figure 8:**
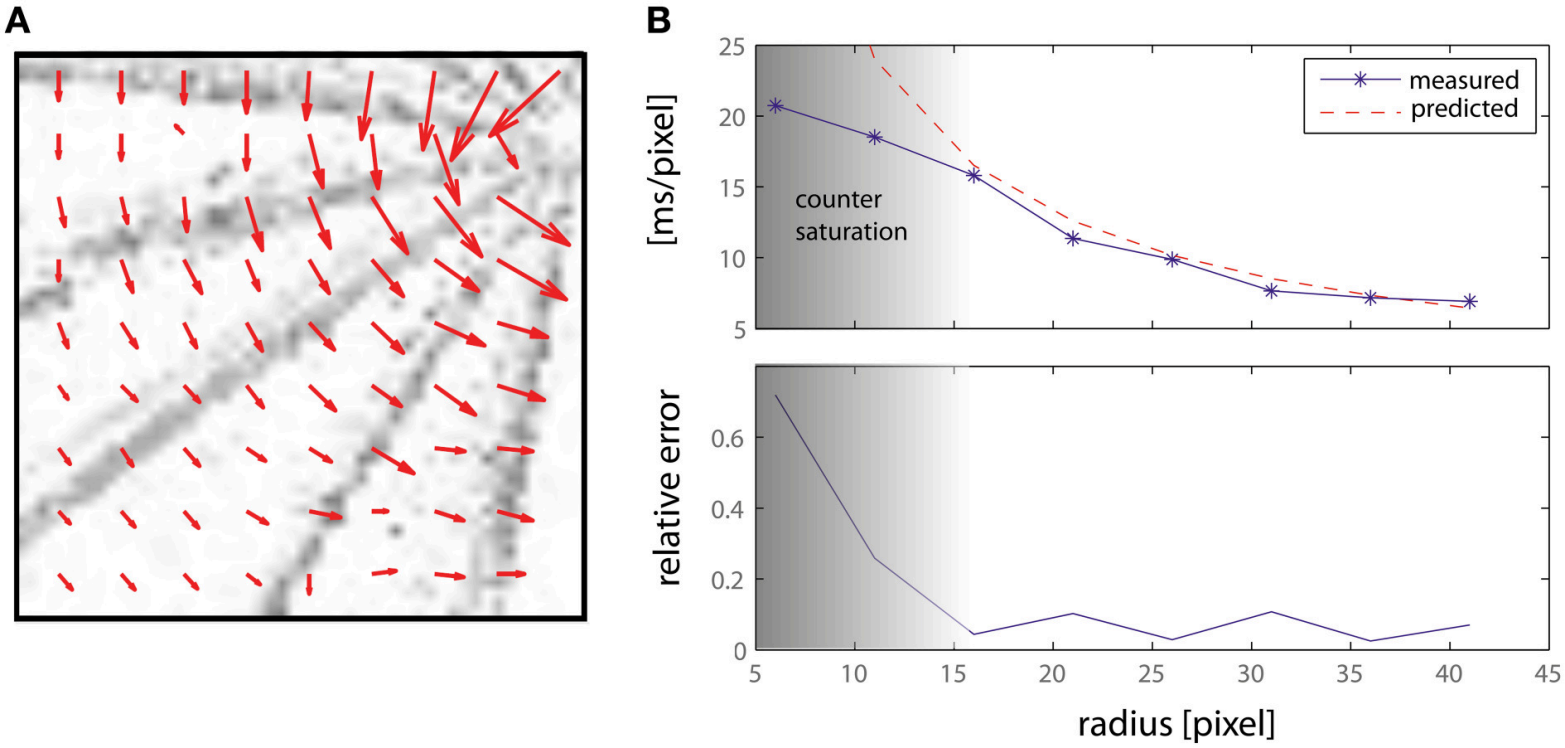
**(A)** Delay vector fields accumulated over time from a rotating bar. Each grayscale *frame* is obtained accumulating retina spikes for 0.1 s. The vector fields have been obtained from an off-line analysis thought to emulate a number of DS detectors larger than those actually implemented on-chip (see text for details). The central detectors have been disabled. **(B)** Measured and predicted speed magnitude, expressed as a delay (ms/pixel), and relative error.

The first experiment shows the motion direction and amplitude of the system while responding to a bar rotating at a constant angular speed ω. Results are presented in Figure 8: the top panel shows the lower left quadrant of the retinal input, with the vector field superimposed so as to show the direction and amplitude of the detected movement. The length of the arrows (i.e., the average number of spikes emitted by the counters) decreases as the speed increases toward the edge of the bar. Each grayscale *frame* is obtained accumulating retina spikes for 0.1 s. The lower part of Figure 8 shows the detected motion amplitude plotted against the theoretically predicted one. It is worth noting that above a delay of 15 ms/pixel we incur in the same saturation limits discussed in the previous section and that can be observed in Figure 7, thus showing the working range of our system.

The last experiment shows the performance of our proposed architecture in a natural environment, by estimating the motion direction of a man jumping from in front of the visual sensor. The whole clip, lasting around 2 s, comprises 409,445 events from the retina, corresponding to about 200 KEv/s and it is available in the Supplementary Material. Parameters have been tuned such that the first reliable trigger spike is obtained up to 50 ms delay from first retina spike to avoid false positives. The first estimation of direction of motion is given after just 3 ms after the trigger spike, corresponding to a frame rate of 300 fps. A reliable output is obtained after 20 ms (equivalent to 50 fps); output is provided with microsecond accuracy. It is worth noting that detection happens in parallel and is transmitted across all the output locations, so as to be able to compute both local and global motion.

**Figure 9:**
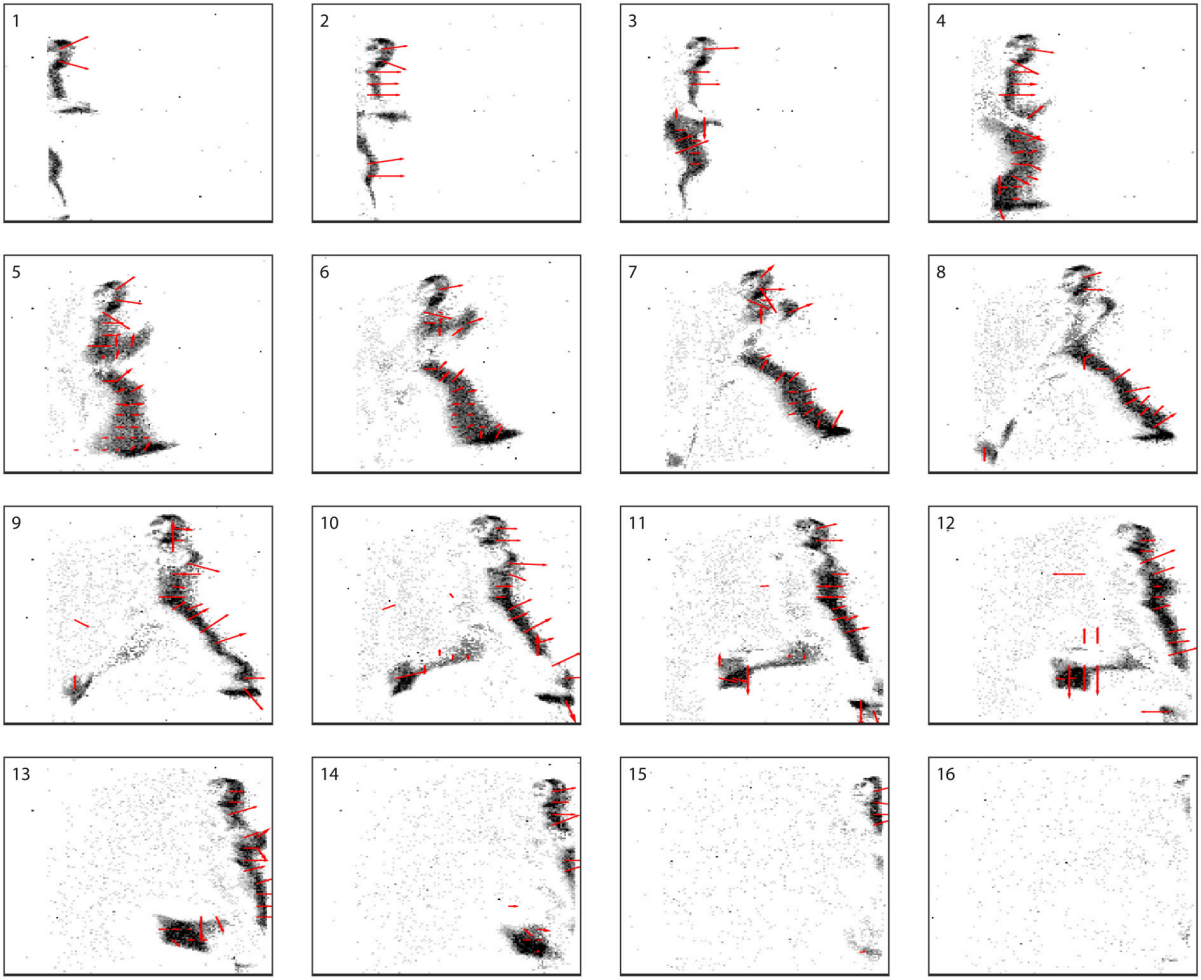
**Sixteen successive frames of a video (available in the Supplementary Material)**. Each frames is obtained by accumulating 50 ms of data. The direction of each arrow indicates the estimated direction of movement as computed by the detector, and the length of the arrow is inversely proportional to the computed speed (longer arrows correspond to slower speeds). As a consequence of the fact that computation is performed asynchronously (not based on a frame time) by the elements in our system, each frame shows an intermediate measurement state for each detector.

Figure 9 shows a series of snapshots, each taken every 50 ms, of both camera events and computation status for the system. The effects of noise can also be observed, for example in frame 10 and 12. The direction of each arrow indicates the estimated direction of movement as seen by the detector, while the length is inversely proportional to the speed estimate (longer arrows correspond to slower speeds). Importantly, as computation is performed asynchronously (not based on a frame time), each panel shows an intermediate measurement state for each detector. A frame-based representation, while offering a visualization in function of time, is not fully adequate to present the results of the system as each detector initiates and terminates a measurement asynchronously.

**Figure 10:**
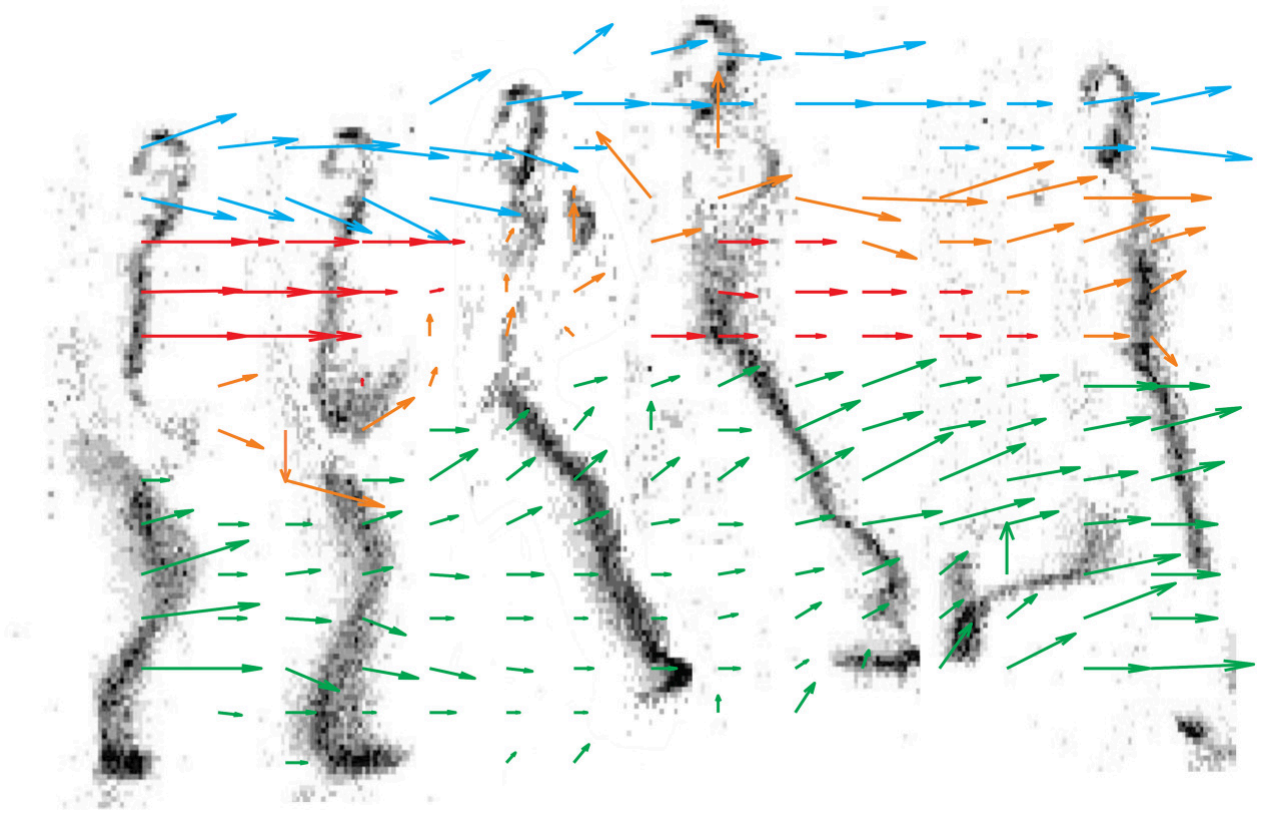
**16 × 16 delay vector field accumulated over time**. Grayscale frames are obtained from 0.1 s of the retina activity. Colors have been introduced by hand just to improve the visualization, no object-segmentation algorithms have been used. Blue vectors track the head, red ones the chest, yellow ones the arm and green is used for the legs.

Figure 10 shows the output of each motion detector independently, and the result of the asynchronous computation is reported by an arrow in the lower panel. As opposed to the previous Figure which samples the state of each detector at given time intervals, in this Figure we have collected the outputs of the detectors as they are produced asynchronously. For each detector we report only its first measurement. While we have not implemented any clustering or segmentation for the output detectors, different colors ease the figure understanding and show how data can be segmented to represent global motion of different objects (in this case different body parts).

## 4. Discussion

This paper has presented a system architecture sensitive to time-of-travels varying over more than 1 order of magnitude. The lower limit is set by the “reaction time” (about 3 ms) of our read-out neurons, the counters (see Section 3.1). The upper limit derives from the longest controllable on-chip inhibitory pulse (about 1 s). This limited range could be seen as a major limitation for our architecture. In literature, similar motion detectors either work on analogous ranges (Kramer, 1996) or are selective to a single speed value (Benson and Delbrück, 1991). In Etienne-Cummings et al. (1997) a different motion detector, implemented with analog differentiators and digital cells, is shown to work over a range of 3 orders of magnitude. Its output is a digital pulse whose duration codes for the time-of-travel. With the on-chip read-out system, an analog integrator for global pixel averaging, the range reduces to 2 orders of magnitude. To the best of our knowledge this is the motion detector with the most extended working range.

We propose a different architecture that, while not reaching such a working speed range, opens to new possibilities for further computational layers. Entirely built from integrate-and-fire neurons, our system output coincides with the bursting activity of the various counters easing implementation of further neural layers for a full local, parallel asynchronous neuromorphic solution. An example consists in cells encoding the time-of-travels in the length of emitted spike bursts, as observed in the rabbit's retina (Barlow and Levick, 1965). Further computational layers could also extend the speed range, as envisaged in what follows.

Up to a certain limit, we can shift the speed range by varying the distance amongst the macropixels, hence changing the size of the motion detector receptive fields. In our architecture this would simply require remapping the retina-to-trigger connection. Adjacent, or even overlapping macropixels could be used in case of slow moving objects, while largely spaced macropixels would be convenient to detect fast motion. One can also imagine, as found in biological systems (Vaney et al., 2001), to design a system of DS units with heterogeneous visual fields. A source of natural heterogeneity in analog systems comes from the mismatch amongst the units, neurons, and synapses. As a result, some units react faster than others. Such property could be exploited to design heterogeneous overlapping “fast” and “slow” motion estimation paths. An extra layer of computation could use this information, leading to an increase of the working range of the proposed architecture.

The speed range of the architecture is linked to the size of the receptive fields of the DS units or, in other terms, by their spatial resolution. In this work we propose a macropixel-to-trigger convergence scheme. Although this could slightly reduce the system resolution, the gained robustness is worth the price. Even though similar convergent schemes are often find in biological systems, to our knowledge no other neuromorphic motion sensor performs analogous local averaging. Robustness in the computation derives also from the inhibition-based algorithm. The correlation or gradient-based methods adopted in other works require quite an accurate tuning of the parameters (see Möckel, 2012 for a review), otherwise accuracy could be severely affected. On the opposite, the scheme proposed by Barlow and Levick is less affected by mismatch since it is just a veto mechanism with a shunting inhibition that should last much more than the excitatory synaptic currents. This is sufficient to obtain reliable results. In our architecture the only critical parameter is the firing rate of the output neurons; without an informed user, all the counters should fire at similar frequencies. We enforced this simply by using a redundant number of excitatory synapses and a small set of three neurons per counter.

Other than biological inspiration and robustness to noise, IF neurons naturally allow to build a specific structure and encode information in the spike timing. Geometry of connections and event timing are the basic elements to perform computation in our architecture. The same elements can be found in the mathematical formulation of the “space of events” introduced in Benosman et al. (2014) where every event is represented as a (x,y,t) tuple. Starting from this “space of events” they propose a fully differential solution for the optical flow computation. Our architecture can be seen as a neuromorphic realization of such a mathematical formulation.

We move from a differential formulation of the problem in the mathematical space of the events, to a hardware neuromorphic network, by encoding the computation critical quantities on ISIs, proposing a neuromorphic network for real-time extraction of the optical flow.

## 5. Conclusion

We demonstrated a fully frame-less autonomous neuromorphic system performing apparent visual motion detection. In this work we have presented an example of how mathematical properties of the event space can be systematically exploited to solve a specific task, optical flow estimation, in neuromorphic hardware. Our architecture is built on few simple ideas supported by specific biological evidences: (1) an inhibition-based mechanism to extract visual motion, (2) convergent many-to-one connections, (3) parallel computation based on precise spike timing, and (4) asynchronous spiking communication. We demonstrated the robustness of this approach to input noise and to circuital mismatch. Using the ATIS retina and the FLANN chips with simple visual scene we demonstrated reliable extraction of the optical flow in real-time. The low computational load and the fast response of the system make it appealing for autonomous robotic application.

## Author contributions

All authors listed, have made substantial, direct and intellectual contribution to the work, and approved it for publication.

### Conflict of interest statement

The authors declare that the research was conducted in the absence of any commercial or financial relationships that could be construed as a potential conflict of interest.
